# FAWPAR at EMOP 2024 and the Special Issue

**DOI:** 10.1016/j.fawpar.2026.e00335

**Published:** 2026-04-21

**Authors:** Olgica Djurković-Djaković, Lucy J. Robertson, Alvin Gajadhar, Ray H. Gamble

**Affiliations:** a University of Belgrade Institute for Medical Research, Belgrade, Serbia; b Faculty of Veterinary Medicine, Norwegian University of Life Sciences, Ås, Norway; c University of Saskatchewan Veterinary Microbiology, Saskatoon, Saskatchewan, Canada; d National Academies of Sciences Engineering and Medicine, Washington, DC, United States

**Keywords:** Food and waterborne parasites, European Multicolloquium of Parasitology (EMOP 2024), FAWPAR sessions, European Federation of Parasitologists (EFP), Diagnostics / detection, Epidemiology

## Abstract

The 14th European Multicolloquium of Parasitology (EMOP), held in Wrocław, Poland in August 2024, marked a significant moment for the European Federation of Parasitologists (EFP), with *Food and Waterborne Parasitology* (FAWPAR) becoming its official journal earlier that year. Dedicated FAWPAR sessions showcased cutting-edge research on transmission, diagnostics, epidemiology, and applied case studies, reflecting the multidisciplinary theme of EMOP 2024. This Special Issue, comprising 15 papers drawn from both the FAWPAR sessions and other EMOP contributions within the journal's scope, highlights methodological innovations, surveillance studies, approaches to addressing applied food-safety challenges, and critical reviews. Together, these contributions underscore the vitality of research in food and waterborne parasitology and reaffirm the importance of international collaboration in addressing global food and health challenges.

## 1 Introduction

The European Multicolloquium of Parasitology (EMOP), the official conference of the European Federation of Parasitologists (EFP), has been held every four years since 1971. Its origins lie in the Cold War era, when parasitologists from both sides of the Iron Curtain sought to overcome political barriers to scientific exchange and cooperation. The founding of the EFP in Poland in 1966, under the leadership of Witold Stefański, and the first EMOP in Rennes, France in 1971, established a tradition of convening parasitologists from across Europe and beyond. That tradition continues today, and, in 2024, EMOP returned to Poland for the second time, hosted in the historic city of Wrocław.

The 14th EMOP was held in late August, bringing together participants from more than 30 countries. The breadth of participation underscored the international scope of parasitology and the continuing relevance of the field. The conference motto, *“Multidisciplinarity in Current Parasitology,”* reflected the inherently interdisciplinary nature of parasitology, which spans human and veterinary medicine, biology, ecology, climate science, and, increasingly, data science and social sciences. In a world marked by uncertainty — geopolitical conflicts, now intensified, climate change, and economic instability — EMOP 2024 nevertheless provided a reassuring continuity: a place where parasitologists could meet, exchange ideas, and reaffirm the importance of international cooperation.

## 2 FAWPAR as the Official Journal of the EFP

A significant development framed EMOP 2024: as of January that year, *Food and Waterborne Parasitology* (FAWPAR) became the Official Journal of the EFP. This formal relationship underscored the growing importance of food and waterborne parasitology within the EFP's remit. It also provided the rationale for dedicating a large part of the EMOP program to FAWPAR, and for publishing a Special Issue (SI) to capture the research presented. The FAWPAR sessions at EMOP 2024 were thus not only scientific highlights but also symbolic of the journal's new role as the EFP's voice in this area.

## 3 FAWPAR sessions at EMOP 2024

Two sessions, FAWPAR I and II, were held on Tuesday, 27 August. Organized and chaired by Lucy Robertson and Olgica Djurković Djaković, they brought together plenary lectures, invited talks, poster presentations, and a panel discussion.

Session I opened with a plenary lecture by Lucy Robertson on determining the human impact of transmission of food- and waterborne parasites to people — a topic that framed the scope of FAWPAR itself. Invited talks followed: Simone Cacciò discussed metagenomic approaches to detect protozoa in food and water; Jeroen Saeij presented on genetic determinants of *Toxoplasma gondii* oocyst resistance; and Rachel Chalmers reviewed lessons learned from *Cryptosporidium* outbreaks. Poster presentations were expansive, covering *Trichinella* infection in Southeastern Europe, unusual food poisoning due to *Anisakis*, synanthropic birds and rodents as indicators of *Toxoplasma* contamination, soil contamination in Poland, and freshwater ecosystems as reservoirs of zoonotic parasites.

Session II continued with invited talks by Ivona Mladineo on fish-borne parasites of importance for the EU, and Adriano Casulli on community risk of cystic echinococcosis, before culminating in a lively panel discussion, in which Joana Firmino from the European Food Safety Authority participated online and provided useful insights on the perspective of that agency regarding food and waterborne parasites. The quality of the invited talks as well as of the following discussion was remarkable, and the sessions as a whole demonstrated the richness of current research in food and waterborne parasitology. Although only some of these presentations were subsequently developed and published in this SI, the sessions provided the inspiration and framework for the collection.

FAWPAR also instituted prizes for the best presentations by students and emerging scientists. At EMOP 2024, the awards were presented to Cecilia Wangari Wambui (Catholic University Leuven, Belgium; paper included in this SI) and Karolina Kot (Pomeranian Medical University in Szczecin, Poland), recognizing their outstanding contributions. These prizes underscored FAWPAR's commitment to supporting excellence and encouraging the next generation of parasitologists.

## 4 The Special Issue

The SI comprises 15 papers, reflecting the diversity of topics covered in the FAWPAR sessions and other EMOP contributions within the journal's scope. Rather than listing them exhaustively, it is useful to group them into thematic categories that mirror the discussions in Wrocław.

• **Diagnostics and methods:** Several papers focus on methodological innovation. These include environmental DNA detection for schistosomiasis, metagenomic approaches to detecting parasites in food and water, and the transformation of a *Cryptosporidium* reference service to meet One Health challenges. Together, they highlight the importance of advancing detection and surveillance tools.
• **Epidemiology and surveillance:** A second group of papers address epidemiological patterns and outbreak investigations. Reviews of *Giardia* and *Campylobacter* outbreaks in Europe, studies of *Enterocytozoon bieneusi* in buffalo calves, *Sarcocystis* detection in farm environments, and *Blastocystis* in marine animals illustrate the breadth of surveillance efforts. These contributions underscore the continuing relevance of food and waterborne parasites in both human and animal health.
• **Applied case studies:** Several papers present case studies with direct implications for food safety. Research on *Anisakis* in fish, including consumer risk assessments and unusual hyperinfection cases, exemplifies the intersection of parasitology with public health and food industry concerns. Studies on fish-borne parasites and echinococcosis likewise highlight applied challenges.
• **Reviews and perspectives:** Finally, the SI includes critical reviews of *Toxoplasma gondii*, *Trichinella* proteins, and transmission routes of parasites via food. These papers provide broader perspectives and syntheses that complement the empirical studies.

Together, these 15 papers reflect both the diversity of the FAWPAR sessions and the wider EMOP contributions. They demonstrate the inherently multidisciplinary nature of parasitology, bridging molecular biology, epidemiology, environmental science, and applied food safety.

## 5 Looking forward

The publication of this SI marks the beginning of a sustained partnership between FAWPAR and the EFP. The dedicated FAWPAR sessions at EMOP 2024, and the subsequent SI, establish a model that will be continued and integrated into future EMOPs. FAWPAR sessions will become a recurring feature of the conference, and the resulting SIs will provide a record of the research presented and opportunities to presenters for publication of their work. In this way, FAWPAR will serve not only as the official journal of the EFP but also as the journal of record for food and waterborne parasitology within the EMOP framework. FAWPAR prizes for students and emerging scientists, introduced at EMOP 2024, will likewise continue at future meetings, further encouraging excellence among the next generation of parasitologists.

At the beginning of 2026, ownership of FAWPAR was formally transferred from its founder, the International Association for Food and Waterborne Parasitology (IAFWP), to the EFP. This development further consolidates the journal's role as the official voice of the EFP in food and waterborne parasitology, and strengthens the foundation for FAWPAR sessions and SIs at future EMOPs.

The importance of food and waterborne parasites is unlikely to diminish. Globalization, climate change, and shifting agricultural and food practices continue to create new challenges. Parasites know no borders, and international cooperation remains essential. The FAWPAR sessions at EMOP 2024 demonstrated the vitality of research in this area, and this SI captures a snapshot of that progress. Future EMOPs will build on this foundation, and FAWPAR will continue to provide a platform for sharing and publishing advances, fostering collaboration, and strengthening our collective response to these enduring as well as emerging challenges.

## 6 Conclusions

The FAWPAR sessions at EMOP 2024 were a highlight of the meeting, and the 15 papers published here reflect both the diversity of those sessions and the wider EMOP contributions relevant to food and waterborne parasitology. It is our hope that this collection will serve both as a record of EMOP 2024 and as a resource for researchers and practitioners working to protect food and water safety. Looking ahead, food and waterborne parasites will remain central to global health, and FAWPAR will continue to provide a platform for the parasitology community to share advances and foster collaboration.

## Declaration of competing interest

The authors declare the following financial interests/personal relationships which may be considered as potential competing interests:

Olgica Djurkovic-Djakovic reports a relationship with European Federation of Parasitologists that includes: president of the Board.

Lucy J. Robertson reports a relationship with European Federation of Parasitologists that includes: general secretary of the Board.

All authors served as editors for this SI. They are also editors of this journal (Olgica Djurkovic-Djakovic is the EiC; Lucy Robertson is an AE; Alvin Gajadhar is the Founding Editor and former EiC, Ray Gamble is a former AE and now member of the Editorial Advisory Board).

